# A New Evolutionary Model for Hepatitis C Virus Chronic Infection

**DOI:** 10.1371/journal.ppat.1002656

**Published:** 2012-05-03

**Authors:** Rebecca R. Gray, Marco Salemi, Paul Klenerman, Oliver G. Pybus

**Affiliations:** 1 Department of Zoology, University of Oxford, Oxford, United Kingdom; 2 Department of Pathology, Immunology & Laboratory Medicine, University of Florida, Gainesville, Florida, United States of America; 3 Nuffield Department of Medicine, University of Oxford, Oxford, United Kingdom; The Fox Chase Cancer Center, United States of America

Hepatitis C virus (HCV) infects an estimated 3% of humanity [1] and is a leading global cause of liver disease and liver cancer [2]. Intervention is currently limited by the lack of a vaccine and of universally successful drug treatments. Although several next-generation drugs (e.g., direct-acting protease-inhibitors) are already improving outcomes, a number of factors will affect overall treatment success [3]. Among these, viral genetic variation and the emergence of drug resistance are of major importance.

It is now recognized that effective prevention and treatment of HCV infection requires an appreciation of the virus' evolutionary behaviour. HCV evolves very rapidly during infection within a host, resulting in a genetically diverse viral population (often termed a “quasispecies”) whose composition is determined by a combination of evolutionary processes that include mutation, replication rate, natural selection, and random genetic drift. As a consequence, patterns of HCV genetic diversity within infected patients (if correctly analysed) should reveal information about the evolutionary dynamics of infection and could uncover links between viral evolution and the progression of clinical disease.

Studies that have followed this logic are too numerous to review here and vary widely in the number and health status of patients investigated, the timespan over which viruses are sampled, and the quality of viral genetic information obtained (ranging from whole viral genomes to heteroduplex mobility assay data). In most studies, HCV genomic sequences are obtained from peripheral blood and are summarized using two measures: the *diversity* of sequences sampled at any one time and the *divergence* among sequences sampled at different times. No clear-cut patterns in these statistics among patients with HCV have emerged. For example, with respect to patient outcomes, high diversity in the HCV envelope region is associated with progression from acute to chronic infection [4], [5] yet also with milder symptoms [6], [7] and possibly with poorer outcomes after drug treatment [8].

It is helpful to interpret these results in the context of evolutionary theory. First, it is well-established that much of the evolutionary information inherent in sequence data is lost when they are compressed into summary statistics such as diversity or divergence [9], [10]. In retrospect, it is perhaps optimistic to expect one or two numerical values to capture much of the complexity of host–viral interactions during infection. Second, and more importantly, theory tells us that an evolutionary *model* is always required to infer the behaviour of a viral population from sequences sampled from it [11], [12]. When previous HCV studies have correlated diversity with patient outcomes, they have, by default, implicitly applied a very simple evolutionary model—one that assumes a single, well-mixed viral population whose members all replicate and evolve identically.

**Figure 1:**
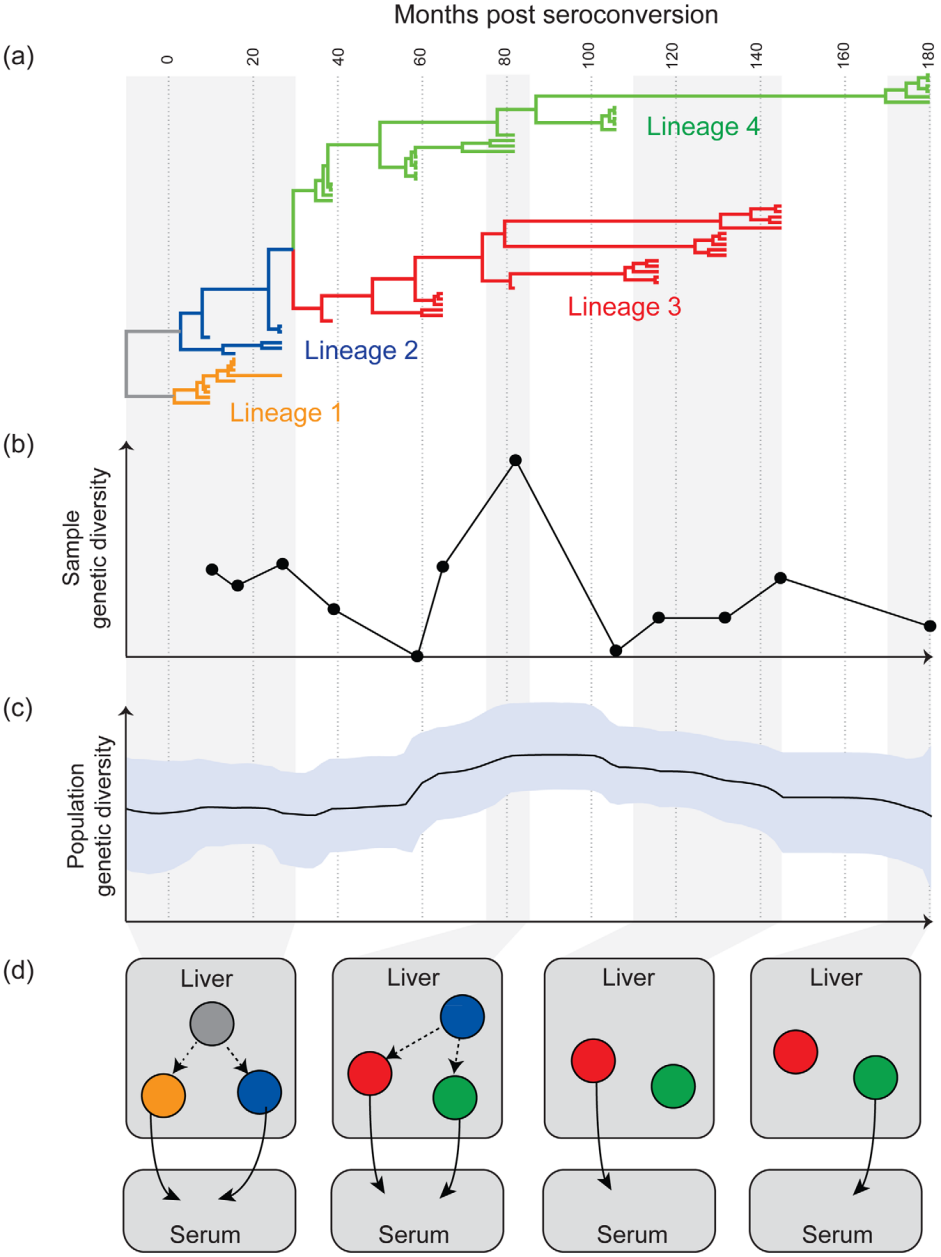
Aspects of the evolutionary dynamics of chronic HCV infection. (a), (b), and (c) show various analyses of HCV gene sequences (E1E2 region) sampled longitudinally from a single infected individual (Pt11 in [7]). Sequences were obtained from serum at twelve occasions over 15 years. All results are placed on the same time scale (top). (a) The phylogeny of the sampled sequences was reconstructed using a molecular clock model, such that branch lengths represent time (calculated using BEAST v1.6.2 [30]). To aid explanation, branches have been grouped into four lineages, indicated by colour. Lineages 3 and 4 co-exist between months 30 and 145. Lineage 4 was present in the patient but undetected at months 65, 116, 132, 145. (b) The average diversity of sequences obtained at each sampling time (mean pairwise genetic distance; calculated using MEGA v4 [31]). The vertical scale represents mean substitutions per site. Sample diversity is notably higher at month 82 because both lineages 3 and 4 are detected. (c) An estimate of the genetic diversity of the whole viral quasispecies through time. This figure was calculated using the Bayesian skyline plot method in BEAST [30], [32], which takes into account both sampled and unsampled lineages. The shaded area shows the 95% uncertainty range around the estimate (solid line). (d) A new evolutionary model of HCV infection, as applied to the patient data shown in (a), (b), and (c). Each cartoon represents the state of infection at a different time, as indicated by the shaded areas. Circles represent different sub-populations of HCV-infected cells within the liver (or other sites of extra-hepatic replication), coloured to correspond to the lineages in (a). Solid arrows indicate the fluctuating detection of HCV lineages in serum through time.

However, phylogenetic analyses of within-patient HCV sequences indicate that this simple model is insufficient to explain HCV evolutionary dynamics during infection. Specifically, phylogenies reconstructed from HCV genomes sampled from multiple time points frequently reveal two or more genetically distinct co-existing viral lineages that are not detected at all sampling times; this is seen in both acute [13] and chronic (e.g., [14]–[17]) infection. Figure 1a presents an illustrative within-patient phylogeny, in which lineages 3 and 4 co-exist for more than 10 years. Crucially, the composition of viruses sampled from peripheral blood varies greatly; at one time point only lineage 3 is detected, whilst at the next only lineage 4 might be seen; at yet other times both lineages are observed. The phylogeny thus explains why simple measures of sample diversity oscillate wildly (Figure 1b). By applying more sophisticated methods (see figure legend and [11]), it becomes possible to estimate the genetic diversity of the *whole* viral population, which is comparatively constant through time (Figure 1c). We therefore suggest that the diversity scores (Figure 1b) commonly used in HCV studies do not fully represent the dynamics of infection.

The behaviour illustrated in Figure 1 requires a more complex model than is commonly assumed. We believe the most important feature missing from current descriptions of HCV dynamics is *population structure*; without this, it is very difficult to explain why viral lineages that remain unobserved for several years do not go extinct before later reappearing (Figure 1). Consequently, we propose a new evolutionary model for HCV, in which the lineages that co-exist during chronic infection represent genetically distinct subpopulations of infected liver cells (Figure 1d; extra-hepatic replication is discussed below). Importantly, this model can reconcile many aspects of HCV within-host genetic data (i.e., the data in Figure 1a–1c).

The observation that only a subset of viral subpopulations is detected in serum at any given time may be explained by one of two modulating mechanisms. First, all viral lineages present in the liver may be shed at a roughly constant rate, but levels of neutralizing antibodies targeting specific epitopes may fluctuate over time, modifying the relative frequency of those lineages in peripheral blood. Second, the viral subpopulations may replicate or shed virus at different rates. Factors that might generate such variation include the effect of host cell type on viral replication dynamics, interaction with interferons, or viral interference in the cell cycle [18]. The presence of replication rate variation is supported by mathematical models of HCV infection kinetics [19] and may help explain why HCV quasispecies exhibit strong heterogeneity in the rate of molecular evolution [20].

The notion of HCV population structure is consistent with a wide variety of experimental data, including evidence for cell-to-cell viral transmission [21], [22], the observation of hepatic foci of infection [23], and HCV genetic variation within the liver [24]. Although our illustration (Figure 1d) represents viral sub-populations existing in different liver locations, the population structure we propose could equally arise from non-hepatic replication, as detected in monocytes/macrophages [25], lymphocytes [26], and brain tissue [27]. These cells often harbor viruses with distinct genetic signatures [28], [29].

The under-appreciated complexity of chronic HCV evolution has several practical consequences. First, simple statistics of viral genetic variation (sample diversity, divergence) will be of limited utility; we instead recommend that analyses begin with a phylogenetic approach. Second, our model suggests that viruses sampled from peripheral blood give an incomplete picture of HCV infection dynamics. Where possible, future studies should incorporate additional sites, including liver biopsies, explant livers, and other cell types. Third, population structure can maintain high viral diversity within a patient even when serum viraemia and diversity are low, perhaps contributing to the evolution of drug resistance and to treatment failure. The emergence of new anti-HCV drugs therefore makes the understanding of HCV evolutionary dynamics a research priority.
